# Enhancement of insulin-mediated rat muscle glucose uptake and microvascular perfusion by 5-aminoimidazole-4-carboxamide-1-β-d-ribofuranoside

**DOI:** 10.1186/s12933-015-0251-y

**Published:** 2015-07-22

**Authors:** Eloise A Bradley, Lei Zhang, Amanda J Genders, Stephen M Richards, Stephen Rattigan, Michelle A Keske

**Affiliations:** Menzies Institute for Medical Research, University of Tasmania, Private Bag 23, Hobart, 7001 TAS Australia; Garvan Institute of Medical Research, Darlinghurst, NSW Australia; Institute of Sport, Exercise and Active Living (ISEAL), Victoria University, Melbourne, VIC Australia; School of Medicine, University of Tasmania, Hobart, TAS Australia

**Keywords:** Muscle, Insulin, Glucose, Microcirculation, Microbubbles

## Abstract

**Background:**

Insulin-induced microvascular recruitment is important for optimal muscle glucose uptake. 5-aminoimidazole-4-carboxamide-1-β-d-ribofuranoside (AICAR, an activator of AMP-activated protein kinase), can also induce microvascular recruitment, at doses that do not acutely activate glucose transport in rat muscle. Whether low doses of AICAR can augment physiologic insulin action is unknown. In the present study we used the euglycemic hyperinsulinemic clamp to assess whether insulin action is augmented by low dose AICAR.

**Methods:**

Anesthetized rats were studied during saline infusion or euglycemic insulin (3 mU/kg/min) clamp for 2 h in the absence or presence of AICAR for the last hour (5 mg bolus followed by 3.75 mg/kg/min). Muscle glucose uptake (R’g) was determined radioisotopically with ^14^C-2-deoxyglucose and muscle microvascular perfusion by contrast-enhanced ultrasound with microbubbles.

**Results:**

AICAR did not affect blood glucose, or lower leg R’g, although it significantly (p < 0.05) increased blood lactate levels and augmented muscle microvascular blood volume via a nitric oxide synthase dependent pathway. Insulin increased femoral blood flow, whole body glucose infusion rate (GIR), R’g, hindleg glucose uptake, and microvascular blood volume. Addition of AICAR during insulin infusion increased lactate production, further increased R’g in Type IIA (fast twitch oxidative) and IIB (fast twitch glycolytic) fiber containing muscles, and hindleg glucose uptake, but decreased R’g in the Type I (slow twitch oxidative) fiber muscle. AICAR also decreased GIR due to inhibition of insulin-mediated suppression of hepatic glucose output. AICAR augmented insulin-mediated microvascular perfusion.

**Conclusions:**

AICAR, at levels that have no direct effect on muscle glucose uptake, augments insulin-mediated microvascular blood flow and glucose uptake in white fiber type muscles. Agents targeted to endothelial AMPK activation are promising insulin sensitizers, however, the decrease in GIR and the propensity to increase blood lactate cautions against AICAR as an acute insulin sensitizer.

## Background

Insulin stimulates skeletal muscle glucose uptake in vivo indirectly by regulating microvascular blood flow [1–4] and directly via insulin receptors on the myocytes resulting in increased GLUT4 translocation to the sarcolemma [5, 6]. The insulin-mediated vascular responses facilitate insulin and glucose delivery to the myocytes and have been shown to occur in both humans [7–10] and experimental animals [1, 3, 11]. The recruitment of microvascular blood flow by insulin is impaired in insulin-resistant rats [12–14] and humans [7, 8]. Loss of insulin-mediated microvascular recruitment in muscle can alone result in whole body insulin resistance equivalent to that caused by obesity [11, 12], emphasizing the physiological importance of this process [15].

Insulin’s hemodynamic actions in vivo appear to be mediated, at least in part, by nitric oxide (NO) dependent processes [16, 17], and local nitric oxide synthase (NOS) inhibition impairs insulin-mediated microvascular recruitment and decreases insulin-mediated skeletal muscle glucose uptake [18]. However, simply delivering NO to the muscle vasculature does not lead to microvascular recruitment or enhance insulin-mediated muscle glucose uptake [19, 20] but appears to require insulin action at specific vascular sites [15, 20, 21].

Insulin has been shown to activate the PI3K/Akt pathway leading to phosphorylation and activation of endothelial nitric oxide synthase (eNOS) and NO production [22, 23]. Inhibition of PI3K by wortmannin in rats in vivo also leads to loss of insulin-mediated microvascular recruitment in muscle suggesting this pathway is important [24]. The phosphorylation and activation of eNOS can also occur by other kinase pathways and we have reported that activation of AMP-activated protein kinase (AMPK) by 5-aminoimidazole-4-carboxamide-1-β-d-ribofuranoside (AICAR) increases NO synthesis and muscle microvascular perfusion [25]. AICAR, at high doses acutely activates glucose transport in resting rat muscle in vitro [26, 27] and in vivo [28], however it is not known if lower doses which do not have a direct metabolic action can sensitize physiologic insulin action. There have been few studies examining the effect of AMPK activation combined with insulin. Further, there have been no studies examining whether AMPK activation by AICAR improves the ability of insulin to increase microvascular perfusion in skeletal muscle. Thus, in the present study we have examined the acute effect of AICAR on muscle glucose uptake in vivo during a physiologic insulin infusion. The dose of AICAR used was sufficient to cause microvascular recruitment but not stimulate muscle glucose uptake alone [25].

## Methods

### Animal care

All procedures adopted and experiments undertaken were approved by the University of Tasmania Animal Ethics Committee and performed in accordance with the Australian Code of Practice for the Care and Use of Animals for Scientific Purposes—2004, 7th Edition. Male Hooded Wistar rats weighing 243 ± 1 g at the time of the experiments were obtained from the University of Tasmania Animal House (Hobart, Australia). Animals were raised on a commercial diet (Pivot, Launceston, Australia) containing 21.4% protein, 4.6% lipid, 68% carbohydrate and 6% crude fiber with added vitamins and minerals together with water ad libitum and were housed at 21 ± 1°C on a 12:12 h light:dark cycle.

### Surgery

Complete details are as described previously [1]. Briefly, rats were anesthetized and cannulas surgically implanted into a carotid artery for arterial sampling and measurement of blood pressure, and into both jugular veins for continuous administration of anesthetic and other intravenous infusions. Animals were allowed to spontaneously breathe room air through a tracheostomy tube. Animals were maintained at 37°C under anesthesia for the duration of the experiment using a continual infusion of sodium pentobarbital (0.6 mg/kg/min) via the left jugular cannula. Once the surgery was completed, a period of equilibration of approximately 60 min was allowed so that cardiovascular parameters could become stable and constant.

### Experimental protocols

**Figure 1:**
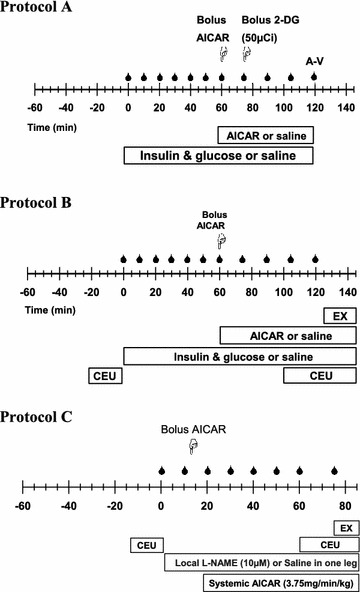
Animals were subject to a 2 h euglycemic clamp of 3 mU/kg/min insulin or saline infusion with or without AICAR. Arterial blood samples (*droplets*) were taken for glucose and lactate analysis. A bolus injection (i.v.) of AICAR (20 mg/kg) is indicated by *pointing hand* and followed by AICAR infusion (3.75 mg/min/kg). Other bolus infusions are indicated by *pointing hand*. In protocol A, arterial and venous blood samples were taken (indicated as A–V) for plasma glucose determination as well as arterial insulin and AICAR determination. The gastrocnemius group of muscles was freeze clamped at 120 min for the determination of AICAR and ZMP content as well as R’g determination. In protocol B, microbubble infusion (40 µL/min) and periods where ultrasound measurement of MvV and MvP were made are shown by CEU. Muscle contraction (field stimulation, 2 Hz, 0.1 ms, 30–50 V) is indicated by EX. In protocol C L-NAME (NOS inhibitor) was infused 15 min prior to and during AICAR infusion.

All animals underwent one of the three following protocols in Figure 1.

#### Procotol A: metabolic and hemodynamic actions of AICAR

Rats were randomly assigned into four experimental groups: (1) saline, n = 33 (2) AICAR (3.75 mg/min/kg, Sigma-Aldrich., St. Louis, MO, USA), n = 16 (3) insulin (3 mU/min/kg, Human R Eli Lilly, Indianapolis, IN, USA), n = 39 or (4) insulin + AICAR (3 mU/min/kg and 3.75 mg/min/kg respectively), n = 18.

AICAR administration was initiated by an intravenous bolus injection (20 mg/kg) at t = 60 min followed by a constant infusion (3.75 mg/min/kg, via the jugular vein) for the remainder of the experiment. Hyperinsulinemic euglycemic clamps (3 mU/min/kg) were performed in the insulin and insulin + AICAR groups to assess insulin sensitivity. Insulin infusions commenced at t = 0 min and continued until the end of the experiment. In the experiments where rats received insulin, basal blood glucose concentration was maintained (euglycemia) by co-infusion of variable rates of glucose (30% w/v solution) over the course of the experiment.

Epigastric vessels were ligated, and an ultrasonic flow probe (Transonic Systems, VB series 0.5 mm) was positioned around the femoral artery of the right leg just distal to the rectus abdominis muscle. Data for femoral artery blood flow (FBF), heart rate (HR), and blood pressure (BP) were sampled using WINDAQ data acquisition software (DATAQ Instruments).

Blood and plasma samples were collected from the carotid artery (A) and femoral vein (V) for determination of blood and plasma glucose, and blood lactate concentration at the end of the experiment. Hindleg glucose uptake was calculated from the arterio-venous difference (A–V) multiplied by the FBF (the Fick Principle) and expressed as µmol/min. The calf muscles (gastrocnemius, soleus and plantaris) were removed from the right leg and freeze clamped in situ at the conclusion of the experiment and stored at −80°C for later determination of AICAR and 5-Aminoimidazole-4-carboxamide-1-β-d-ribofuranosyl 5′-monophosphate (ZMP) content. Both of these metabolites were measured because AICAR is taken up by cells and converted to its active form ZMP.

Muscle glucose uptake was also assessed using isotopic tracers. A intravenous bolus dose (50 µCi, 0.2 ml) of 2-deoxy-d-[1-^14^C]glucose (2-DG), specific activity 1.85-2.29 GBq/mmol; Amersham Life Science, Castle Hill, NSW, Australia) was given 45 min before the end of the experiment. The plasma specific activity of 2-DG was determined from plasma samples (25µL) collected at 5, 10, 15, 30 and 45 min after administration of the 2-DG bolus. At the conclusion of the experiment, individual muscles [soleus, plantaris (Plant), gastrocnemius red (GR), gastrocnemius white (GW), extensor digitorum longus (EDL) and tibialis (Tib)] from the lower left hindlimb were rapidly removed, frozen in liquid nitrogen and stored at −80°C until assayed for 2-DG radioactivity and the determination of muscle specific glucose uptake as described previously [18].

### Effect of fasting on AICAR-mediated metabolic actions

A sub-set of insulin + AICAR experiments were performed using a modified version of protocol A, to assess effect of fasting on AICAR-mediated metabolic actions. These experiments were carried out in order to determine the effect of AICAR on insulin-mediated metabolic responses with different degrees of liver glycogen content (i.e. prolonged fasting depletes liver glycogen). All rats underwent insulin + AICAR (3 mU/min/kg and 3.75 mg/min/kg, respectively) infusion. Rats were randomly assigned into two experimental groups: (1) overnight fast (which represents abstaining from food for 16 h), n = 12 or (2) prolonged fast (which represents abstaining from food for 40 h), n = 16. Whole body GIR and muscle specific glucose uptake (by isotopic tracer) was performed as described above.

#### Protocol B: muscle microvascular actions of AICAR

The effect of AICAR on microvascular perfusion in skeletal muscle was assessed. These measures could not be conducted in Protocol A because microbubbles interfere with the Doppler signal from the Transonic flow probe used to measure FBF.

Rats were randomly assigned into four experimental groups: (1) saline, n = 6 (2) AICAR (3.75 mg/min/kg, Sigma-Aldrich., St. Louis, MO, USA), n = 6 (3) insulin (3 mU/min/kg, Human R Eli Lilly, Indianapolis, IN, USA), n = 6 or (4) insulin + AICAR (3 mU/min/kg and 3.75 mg/min/kg respectively), n = 6.

AICAR, insulin or saline infusion was identical to the procedure described in protocol A. Muscle microvascular perfusion was assessed by contrast-enhanced ultrasound (CEU) as described previously [2].

CEU imaging occurred at baseline (t = 0 min) and at the end of the experiment (t = 120 min) to assess microvascular responses to saline, AICAR, insulin and Insulin + AICAR. Contraction is a well-accepted stimulus for maximal microvascular recruitment. At the end of the experiment, an incision was made through the skin at the lateral side of the hip. Electrodes were attached to the muscle of the hindleg and the Achilles tendon. Twitch contraction was performed with 0.1 ms pulses of 30–50 V. Microvascular responses to contraction were assessed by CEU at the time indicated (Figure 1b).

### CEU imaging

The adductor magnus and semimembranosus muscles of the left hind limb were imaged in short axis with a linear array transducer (L7-4), secured in position for the duration of the experiment, connected to an ultrasound system (HDI-5000, Ultrasound, Phillips Ultrasound). The acoustic focus was set at the mid-muscle level and gain settings were optimised and held constant throughout the experiment. Albumin microbubbles (Optison™, GE Healthcare) were diluted 1:5 with perfluoropropane gassed saline and infused via the jugular vein at 40 µL/min for the duration of the data acquisition. The acoustic signal that is generated from the microbubbles exposed to ultrasound is proportional to the concentration of microbubbles within the volume of tissue being imaged. All microbubbles within the ultrasound beam are simultaneously imaged and destroyed in response to a single pulse of high-energy ultrasound. As the time between successive pulses is prolonged, the beam becomes progressively replenished with microbubbles (refer to Figure in [2]). The beam will eventually become fully replenished with microbubbles, and further increases in the time between each ultrasound pulse will not affect the microbubble signal in tissue. Intermittent imaging was performed using pulsing intervals (PIs) ranging from 0.5 to 15 s to allow incremental microvascular replenishment with microbubbles between each pulse until the volume within the beam was completely refilled. Several frames were obtained at each PI. Data was analysed using QLAB™ Software (Version 6.0, Phillips Ultrasound, Bothwell). The ultrasound intensity in decibels within the region of interest (semimembranous and adductor magnus) were converted to acoustic intensity and after background subtraction using 0.5 s ultrasound images (to eliminate signal from larger blood vessels and muscle per se), a pulsing interval (time) versus acoustic-intensity curve was plotted. This allowed calculation of microvascular volume (MvV) as well as an index of microvascular perfusion (MvP) according to the equation y = A (1 − e^−β(*t*−0.5)^) where y is the acoustic intensity at a given pulsing interval, A = MvV, and Axβ = MvP [2, 29].

#### Protocol C: role of NOS on AICAR-mediated microvascular actions

We have previously demonstrated that insulin [4], but not contraction [30], stimulate microvascular recruitment in muscle via NOS-dependent pathway. We wanted to determine whether AICAR-mediated microvascular recruitment was similar to insulin or contraction. The femoral artery, femoral vein and nerve were all carefully separated. In one leg (test leg), the epigastric artery was cannulated for infusion of N^ω^-l-nitro-arginine-methyl ester (l-NAME, Sigma-Aldrich, St. Louis, MO, USA) to achieve 10 μM, and in the contralateral leg (control leg), the epigastric artery was ligated. Rats were randomly assigned into two groups as follows; (1) AICAR (3.75 mg/min/g), n = 9, or (2) AICAR + local L-NAME, n = 9 (Figure 1c). We have previously demonstrated that this dose of l-NAME supresses insulin-mediated microvascular recruitment while avoiding systemic effects on blood pressure and heart rate [18]. Microvascular responses were assessed by CEU at baseline (t = 0 min) and after AICAR or AICAR + l-NAME infusion. Similar to Protocol B, all animals underwent a bout of acute contraction to assess maximal microvascular recruitment.

### Analytical methods

Blood glucose, plasma glucose and blood lactate concentrations were determined using a glucose analyzer (Yellow Springs Instruments, Model 2300 Stat plus). Plasma insulin concentrations were determined by ELISA (Mercodia, Uppsala, Sweden) from arterial plasma samples taken at beginning and the conclusion of the experiment. AICAR and ZMP concentrations were determined from perchloric acid treated plasma or homogenized muscle samples, that were centrifuged for 10 min and the supernatant analysed using reverse-phase HPLC as generally used to resolve nucleoside mixtures [25, 31].

### Statistical analysis

All data are presented as mean ± SE. Repeated-measures two-way ANOVA was used to determine if there were differences between treatment groups over the time course of the experiment, or one-way ANOVA was used for single point measurements. When a significant difference (p < 0.05) was found, pair wise comparisons by the Student–Newman–Keuls test was used to determine treatment differences. Comparisons between the treatments for plasma AICAR values were determined by an unpaired *t* test. All tests were performed using SigmaStat™ (Systat Software, Inc., San Jose, CA, USA).

## Results

### Procotol A: metabolic and hemodynamic actions of AICAR

#### Effect of AICAR infusion on insulin and blood glucose levels

**Figure 2:**
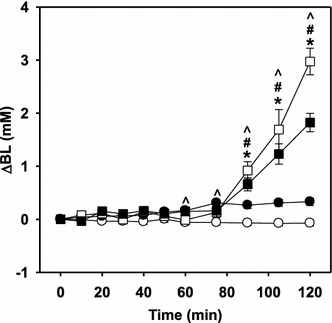
Changes in arterial blood lactate (∆BL) in response to saline (*unfilled circle*), insulin (*filled circle*), AICAR (*unfilled square*) and AICAR + insulin (*filled square*) treatments. Values are given as mean ± SE. *Asterisk* AICAR treated significantly different (p < 0.05) from corresponding untreated animals. *Hash* Insulin + AICAR treated significantly different (p < 0.05) from insulin treated animals. *Cap symbol* Insulin treated significantly different (p < 0.05) from saline treated animals. (Protocol A); 2-way repeated-measures ANOVA.

We first determined whether AICAR infusion alters insulin levels achieved during clamps, to exclude the possibility that alterations in glucose handling result solely from altered plasma insulin concentration. Plasma insulin concentration was significantly elevated (twofold, p < 0.05) following insulin clamps (saline, 204 ± 28 pM; insulin, 410 ± 34 pM) but this was not affected by AICAR (AICAR, 234 ± 52 pM; insulin + AICAR, 425 ± 29 pM). Blood glucose concentration was also unaffected by AICAR infusion during insulin clamps, and was maintained at fasting levels with no differences between groups (saline, 3.49 ± 0.12 mM; insulin, 3.75 ± 0.12 mM; saline + AICAR, 3.41 ± 0.14 mM; insulin + AICAR, 3.41 ± 0.10 mM). However, blood lactate concentration significantly increased twofold (p < 0.05) with insulin (saline, 0.44 ± 0.04 mM; insulin, 0.87 ± 0.09 mM) and this was further enhanced with AICAR (AICAR, 3.52 ± 0.27 mM; insulin + AICAR, 2.46 ± 0.15 mM; Figure 2).

#### Effect of insulin on AICAR metabolism

In contrast to the lack of effect of AICAR on insulin, the converse was not true. Insulin infusion moderately (22%, p < 0.05) lowered plasma AICAR content (AICAR, 266 ± 12 mM; insulin + AICAR, 208 ± 12 mM), possibly due to a stimulating effect of insulin on whole body AICAR clearance. This did not appear to be due to altered hindleg AICAR uptake (arterio-venous difference × flow), which was not significantly different between insulin (76.3 ± 10.7 nmol/min, n = 12) and saline (69.6 ± 5.5 nmol/min, n = 10) treated animals. Similarly, the concentration of AICAR and its metabolite ZMP in individual lower leg muscles was not significantly altered by insulin (Table 1). Despite this, both AICAR and ZMP content in soleus was significantly increased compared to gastrocnemius red (GR) and gastrocnemius white (GW) muscle in the AICAR group, although these differences became non-significant in the insulin + AICAR group.

**Table 1:** Muscle content of AICAR and ZMP

	AICAR (n = 7)		Insulin + AICAR (n = 4)	
	AICAR	ZMP	AICAR	ZMP
	(mmol/g wet wt)		(mmol/g wet wt)	
Soleus	161 ± 9	325 ± 31	152 ± 22	237 ± 33
Plantaris	116 ± 11*	252 ± 29	116 ± 17	207 ± 43
GW	98 ± 16*	170 ± 27*	86 ± 12	145 ± 25
GR	86 ± 7*	174 ± 30*	110 ± 13	188 ± 23

Muscle content of AICAR and ZMP from AICAR and insulin + AICAR treated animals determined at t = 120 min. Values are mean ± SE.

*GW* Gastrocnemius white, *GR* Gastrocnemius red.

* AICAR and ZMP values significantly different (p < 0.05) from soleus values.

#### Effect of AICAR on whole body and muscle glucose uptakes

**Figure 3:**
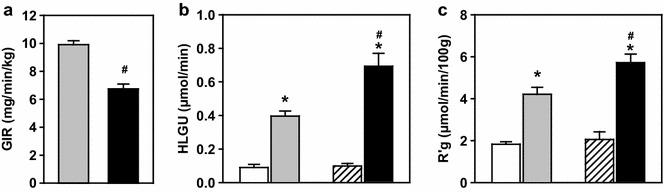
Glucose infusion rate (**a**; GIR), hindleg glucose uptake (**b**; HLGU) and calf muscle specific glucose uptake (**c**; R’g) during saline (*white*), insulin (*grey*), AICAR (hatched) and AICAR + insulin (*black*) treatments. Data are mean ± SE, (Protocol A). *p < 0.05 insulin vs respective saline, ^#^p < 0.05 insulin vs AICAR + insulin.

AICAR reduced (p < 0.001) the glucose infusion rate (GIR) required to maintain blood glucose during the insulin clamp (9.9 ± 0.3 to 6.7 ± 0.3 mg/min/kg during the last 60 min; Figure 3a). No exogenous glucose was required to maintain blood glucose during the AICAR infusion confirming that this dose of AICAR does not have a direct metabolic effect.

Insulin significantly stimulated hindleg glucose uptake (HLGU, fourfold, p < 0.05) and this was augmented by AICAR (p < 0.001, sevenfold; Figure 3b). There was no significant difference between the saline and AICAR groups on HLGU, indicating that AICAR alone had no effect on glucose uptake. Similarly insulin increased muscle glucose uptake (R’g) when assessed by isotopic glucose tracer, and AICAR further enhanced insulin-mediated R’g (p < 0.001, threefold, Figure 3c).

**Figure 4:**
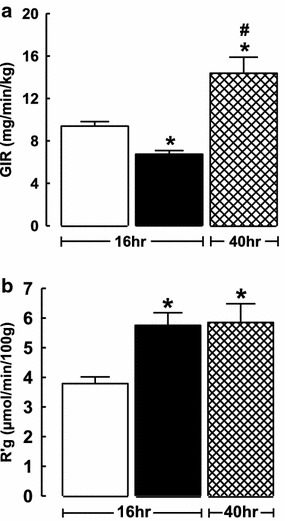
Effect of insulin (*unfilled square*) and insulin + AICAR (*filled square*) [16 h fasted animals] and insulin + AICAR (*crossed square*) [40 h fasted animals] treatment on glucose infusion rate (GIR, **a**) and hindleg 2-deoxyglucose uptake (R’g, **b**). Values were determined at the end of the experiment (Protocol A) and are mean ± SE. *Asterisk* AICAR treated significantly different (p < 0.05) from corresponding untreated animals; *hash* 40 h fast treated significantly different (p < 0.05) from corresponding 16 h fasted group (Protocol A); one-way ANOVA.

The AICAR-mediated decrease in GIR in the presence of increased muscle glucose uptake suggested that AICAR stimulated glucose production from glycogen stores in the liver. This was confirmed in a subset of experiments (Figure 4) in which rats were fasted for 40 h to deplete liver glycogen stores and then were treated with AICAR and insulin. GIR in these 40 h fasted AICAR + insulin rats was significantly greater than insulin alone (Figure 4) indicating that AICAR does not impair GIR in glycogen depleted rats.

**Figure 5:**
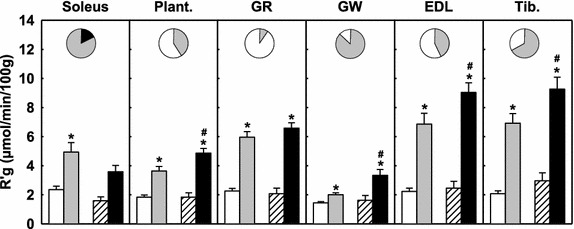
Muscle specific glucose uptake (R’g) for soleus, plantaris (plant.), gastrocnemius red (GR), gastrocnemius white (GW), extensor digitorum longus (EDL) and tibialis (Tib.) muscles during saline (open), insulin (*grey*), AICAR (*hatched*) and AICAR + insulin (*black*) treatments. Percent (%) fiber type is indicated by pie charts; Type I (slow twitch oxidative, (*black*); Type IIA (fast twitch oxidative, *grey*); Type IIB (fast twitch glycolytic, *white*). Data are mean ± SE, (Protocol A). *p < 0.05 insulin vs respective saline, ^#^p < 0.05 insulin vs AICAR + insulin.

Figure 5 shows in a sub-group of experiments, the R’g of individual muscles of the lower leg. Insulin alone significantly stimulated R’g in all muscles of the lower leg. AICAR infusion alone had no significant effects on R’g in any of the lower leg muscles. However, AICAR had disparate effects on the R’g in muscles of the lower leg in the presence of insulin. In the soleus, which is predominately fiber type I (slow twitch oxidative) [32], and the GR, which has a mix of type I and IIA (fast twitch oxidative) fibers [32], there was no significant effect. In contrast, AICAR significantly increased insulin-mediated R’g in the muscles with a mixture of type IIA and IIB (fast twitch glycolytic) fibers [32], plantaris, GW, EDL and tibialis (Figure 5). Since these latter muscles make up the bulk of the muscle in the lower leg the overall effect of AICAR was a significant increase in insulin-mediated glucose uptake (Figure 5).

#### Effect of AICAR on hemodynamic measurements

**Figure 6:**
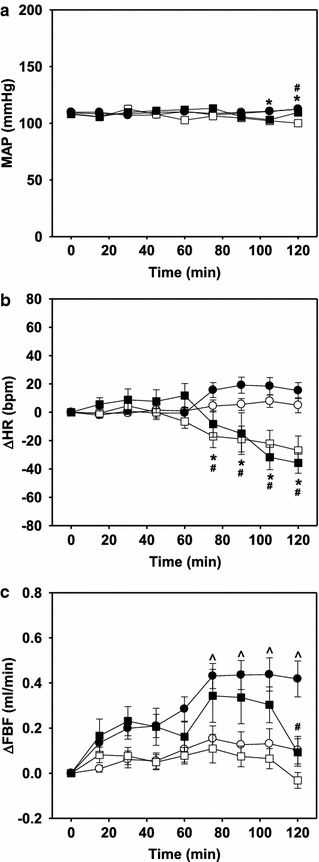
Mean arterial pressure (**a**; MAP), change in heart rate (**b**; ∆HR) and change in femoral artery blood flow (**c**; FBF) during saline (*unfilled circle*), insulin (*filled circle*), AICAR (*unfilled square*) and AICAR + insulin (*filled square*) treatments. Values are given as mean ± SE (Protocol A). *Asterisk* AICAR treated significantly different (p < 0.05) from corresponding untreated animals. *Hash* AICAR + insulin treated significantly different (p < 0.05) from corresponding untreated animals. *Cap symbol* Insulin treated significantly different (p < 0.05) from saline animals; 2-way repeated-measures ANOVA.

Basal mean arterial pressure in anesthetized rats was not affected by insulin or AICAR and remained constant throughout the experiment for all groups (Figure 6a). While insulin did not alter heart rate, AICAR infusion significantly (p < 0.05) reduced heart rate after the first 15 min of AICAR in both the presence and absence of insulin, and remained depressed until the conclusion of the experiment (Figure 6b). Despite the drop in heart rate, AICAR did not significantly alter the increase in femoral artery blood flow (FBF) in response to insulin infusion, which became significant by t = 75 min (Figure 6c). The FBF in the AICAR alone group was not significantly different from the saline control.

### Protocol B: muscle microvascular actions of AICAR

**Figure 7:**
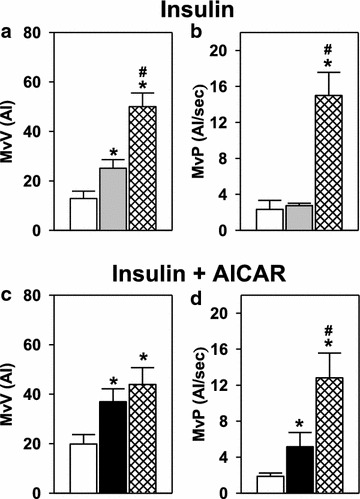
Microvascular volume (MvV) (**a** and **c**) and microvascular perfusion (MvP) (**b** and **d**) for basal (*white*), insulin (*grey*), insulin + AICAR (*black*) and contraction (*cross hatched*). Values are mean ± SE (Protocol B). *p < 0.05 vs basal; #p < 0.05 contraction vs untreated.

Although AICAR did not alter arterial flow, it did enhance insulin-mediated muscle microvascular volume (MvV; Figure 7c) compared with insulin alone (Figure 7a). Indeed, while insulin alone produced the expected increase in MvV, the insulin + AICAR combination produced similar MvV values comparable to that produced by contraction (Figure 7c), which produces maximal MvV in muscle. Insulin infusion had no effect on microvascular perfusion (MvP), although as expected MvP significantly increased during contraction (Figure 7b). Insulin + AICAR treatment significantly increased MvP compared to basal which was further significantly increased with contraction (Figure 7d).

**Figure 8:**
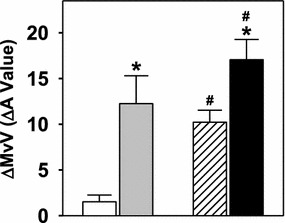
Change in microvascular volume (ΔMvV,) for saline (*white*), insulin (*grey*), AICAR (*hatched*) and AICAR + insulin (*black*) treatments. Data are mean ± SE, (Protocol B). *p < 0.05 insulin vs respective control, ^#^p < 0.05 AICAR vs respective control.

Figure 8 compares the increases in MvV over time between saline, AICAR, insulin and AICAR + insulin treatments. The ΔMvV in the AICAR + insulin group was significantly higher than that of insulin alone, indicating that the effects of AICAR and insulin on microvascular volume are additive.

### Protocol C: Role of NOS on AICAR-mediated microvascular actions

**Figure 9:**
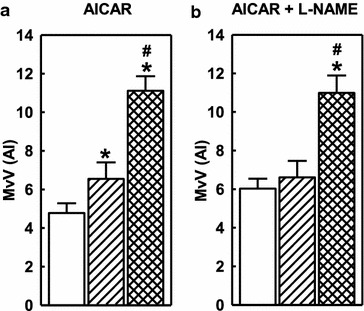
Effect of L-NAME on MvV during baseline (*white*), AICAR (*hatched*), and AICAR + contraction (*cross hatched*). Data are mean ± SE (Protocol C). *Asterisk* Significantly different (p < 0.05) from basal values; *hash* contraction treated significantly different from untreated; 2-way repeated-measures ANOVA.

Microvascular volume recruitment by AICAR was also demonstrated to be NOS-dependent (Figure 9). AICAR increased MvV by 40% (Figure 9a) and this was completely abolished by local hindleg infusion of the NOS inhibitor L-NAME (Figure 9b). Contraction stimulated MvV by 110% and this was unaffected by NOS inhibition.

## Discussion

This study was undertaken to investigate the effects of the AMPK activator AICAR on insulin-mediated microvascular perfusion and glucose uptake in skeletal muscle. The main findings were (1) acute administration of AICAR in vivo, at a dose which does not stimulate glucose uptake by itself, improves insulin-mediated glucose uptake; (2) AICAR induced increases in insulin-mediated muscle glucose uptake which were greater in the Type IIb fiber type muscles; (3) AICAR increases MvV in skeletal muscle by a NOS-dependent pathway; and (4) AICAR augments insulin-stimulated MvV in skeletal muscle.

The effects of insulin to increase total blood flow and MvP in skeletal muscle have been well established [21] and were also observed in the present study. We have previously reported [25] that AICAR, at the dose used in this study, acutely increases MvV and MvP which was also confirmed in the current study. Plasma concentrations of AICAR reached approximately 0.24 mM and although these treatments alone were without effect on muscle glucose uptake, they were found to markedly sensitize insulin action on muscle glucose uptake. Bergeron et al. [26] demonstrated that the effects of AICAR on glucose transport activity are additive with insulin. The results from the present study differ from Bergeron as a lower dose of AICAR was used which was below the threshold to stimulate glucose uptake, despite insulin-mediated glucose uptake being markedly augmented. It is possible that increased insulin mediated glucose uptake in the presence of low dose AICAR is due to increased availability of glucose and insulin to muscle through enhancement of nutritive blood flow. It has been shown that there is a strong correlation between nutritive blood flow and glucose uptake in the presence of insulin [21], and other agents that enhance microvascular perfusion (e.g. methacholine) have been shown to enhance insulin-mediated muscle glucose uptake [20]. This study demonstrated that AICAR-mediated microvascular recruitment is NOS-dependent. This is important because we have similarly shown insulin to recruit flow to the microcirculation via a NOS-dependent pathway. This is not unexpected because activation of AMPK stimulates eNOS and therefore NO production in human aortic cells [33]. This indicates that AICAR and insulin have similar downstream actions leading to NO production and microvascular recruitment in skeletal muscle. Importantly, in the current study L-NAME was infused locally into the hindleg of the rat, therefore AICAR-mediated microvascular actions are due to local muscle NOS and not systemic or central actions.

In the current study AICAR enhanced insulin-mediated increases in skeletal muscle glucose uptake which were found to be dependent upon the fiber type composition of the muscles. Type I (slow twitch oxidative) muscles showed a decrease in insulin-mediated glucose uptake while the type IIA (fast twitch oxidative) and IIB (fast twitch glycolytic) muscles showed a marked increase in insulin-mediated glucose uptake. The fiber type specific effects of AICAR have also been reported by others at higher doses than used in the current study. Bergeron et al. [26] reported AICAR (0.58 mM, in vivo) to directly stimulate 2-DG uptake, in the absence of insulin, in the lateral and medial gastrocnemius and the soleus muscle. Iglesias et al. [34] reported that subcutaneous injection of 250 mg/kg AICAR to insulin resistant high fat fed rats 1 day before conducting a hyperinsulinemic euglycemic clamp markedly improved insulin-stimulated glucose uptake (2-DG) in white but not red quadriceps muscles. Follow up studies by Iglesias et al. [35] also revealed that significant enhancement of insulin stimulated glucose uptake in white muscle also occurred in normal rats. Ai et al. [36] also reported that 0.5–4 mM AICAR stimulated 2-DG uptake occurred in incubated epitrochlearis, less in flexor digitorum brevis and not at all in soleus muscle. Of these muscles, the epitrochlearis is richest in type IIB, the soleus in type I and the flexor digitorum brevis in type IIA fibers [36]. Ai et al. [36] also reported AMPKα1 expression to be highest in the epitrochlearis and lowest in the soleus; flexor digitorum brevis muscles had the lowest expression of AMPKα2. In contrast, Putman et al. [37] demonstrated that the α1 and α2 isoforms of AMPK are consistently expressed in a fiber type specific manner. They showed that both the α1 and α2 protein levels were greatest in the slow-twitch oxidative soleus, slightly less in the mixed fast-twitch red gastrocnemius, and lowest within the fast-twitch white gastrocnemius.

The in vivo findings of this study provide evidence that AICAR increases insulin-mediated hindleg glucose uptake and 2-DG uptake. Despite this finding there was no increase in whole body glucose infusion rate (GIR). The GIR during the insulin + AICAR infusion was significantly reduced, such that by the end of the experiment the rate was approximately 30% less than during infusion of insulin alone. This implies that AICAR inhibits the ability of insulin to suppress hepatic glucose output (HGO). A number of studies have provided in vivo evidence for AICAR inhibiting insulin induced suppression of HGO. A study by Peneck et al. [38] using conscious dogs, showed that intra-portal AICAR infusion of 1 mg/min/kg abolished net hepatic glucose uptake and infusion of 2 mg/min/kg stimulated net HGO. In another in vivo study by Camacho et al. [39] 1 mg/min/kg AICAR was infused into dogs for 90 min during a 2 mU/min/kg hyperinsulinemic insulin clamp. AICAR caused a stimulation of both HGO and hepatic glycogenolysis even in the presence of physiological insulin. Another similar study by Camacho et al. [40] revealed that AICAR is effective in countering the suppressive effect of pharmacological insulin on net HGO which occurred even when AICAR-stimulated Acetyl CoA Carboxylase (ACC) phosphorylation was completely blocked by insulin. These findings indicate that, when activated, AMPK increases the availability of blood glucose to skeletal muscle by countering insulin induced inhibition of hepatic glucose production. Thus AICAR induced HGO provides a reasonable explanation for the observed decrease in GIR during AICAR + insulin treatment in this study in the presence of a net increase in skeletal muscle glucose uptake.

An additional set of experiments were performed with animals fasted for 40 h in which liver glycogen was depleted. In these animals a significantly increased GIR was required to maintain euglycemia during the AICAR + insulin treatment compared with the corresponding overnight fasted treatment, while muscle glucose uptake (R’g), was unchanged (Figure 4) by the prolonged fasting period. This finding confirms that in overnight fasted animals AICAR inhibited insulin’s suppression of HGO.

This study demonstrated that AICAR treatment had two potential detrimental actions in vivo. Firstly, AICAR treatment increased lactate production in resting muscle. This finding is in agreement with other reports which have also observed an increase in lactate concentration with AICAR treatment in vitro [41, 42] and in vivo [26, 39, 43]. Merrill et al. [42] using the perfused rat hindlimb, demonstrated that AICAR treatment (2 mM) increased lactate production by approximately twofold over 45 min compared with the control group. Bergeron et al., [26] using conscious rats, also reported a marked increase in plasma lactate to about 13 mM at 90 min when the plasma levels of AICAR had reached 2.5 mM. Camacho et al. [39] also reported that AICAR significantly increased net hepatic lactate output in the presence of hypoglycemia. The increased blood lactate concentration in AICAR treated animals was also noted by Winder [44]. They suggested that AICAR increased intra-muscular glucose concentration, and the glucose was shunted towards lactate production rather than being oxidised. A number of studies have also demonstrated that AMPK phosphorylates phosphofructokinase and thereby stimulates glycolysis [45–47] and this action is likely to account for the observed increase in net hepatic lactate production. In addition, AICAR treatment has been reported to suppress heart rate, which is most likely due to actions via adenosine receptors in the heart [25, 48]. Although AICAR may have detrimental actions in vivo, the current project does highlight that other agents that operate via AMPK (e.g. vaspin [49] or CNX-012-570 [50]) are important to follow-up as potential new drug targets for insulin resistance and type 2 diabetes.

The genetically obese Zucker, and lipid infused rats, both display impaired insulin-mediated glucose transport in skeletal muscle and insulin-mediated MvP is absent [14, 51]. In contrast, contraction-mediated glucose uptake by muscles in both of these insulin resistant rats appears to be essentially normal as does the increase in MvP in response to exercise [52, 53]. This suggests that the defects particular to insulin signaling may explain peripheral insulin resistance. Bergeron et al. [28] have demonstrated that acute infusion of AICAR in the Zucker rat increases glucose transport during a physiological insulin clamp. The mechanism for enhanced insulin sensitivity in the Bergeron study may be partly due to increased MvP allowing greater access of insulin and glucose to the myocyte. However, in contrast, Lee-Young demonstrated that AICAR infusion into lipid infused mice did not overcome the insulin resistance [54]. It is yet to be determined whether AICAR enhances insulin mediated MvP and thus glucose uptake in models of insulin resistance, such as the rat models described above.

## Conclusions

Low dose AICAR stimulates MvP in skeletal muscle in vivo at a dose which does not stimulate glucose uptake by itself, and enhances insulin stimulated glucose uptake and MvP. The increase in MvP caused by AICAR is likely to enhance hormone and nutrient delivery to the muscle myocytes which in turn increases glucose uptake in skeletal muscle when insulin is present. The AICAR enhanced insulin-stimulated glucose uptake occurred predominantly in the white fiber type muscles. AICAR inhibited insulin’s ability to increase whole body glucose uptake which is likely to be due to inhibition of insulin’s suppression of hepatic glucose output. The stimulation of lactate accumulation and the suppression of heart rate detract from the potential use of AICAR for the treatment of insulin resistance.

## Abbreviations

2-DG: 2-deoxyglucose
AICAR: 5-aminoimidazole-4-carboxamide-1-β-d-ribofuranoside
AMPK: 5′ adenosine monophosphate-activated protein kinase
CEU: contrast-enhanced ultrasound
EDL: extensor digitorum longus
eNOS: endothelial nitric oxide synthase
FBF: femoral artery blood flow
GIR: glucose infusion rate
GLUT4: glucose transporter 4
GR: gastrocnemius red
GW: gastrocnemius white
HGO: hepatic glucose output
HLGU: hindleg glucose uptake
L-NAME: Nω-l-nitro-arginine-methyl ester
NOS: nitric oxide synthase
MvP: microvascular perfusion
MvV: microvascular volume
NO: nitric oxide
R’g: rate of muscle glucose uptake
ZMP: 5-aminoimidazole-4-carboxamide-1-beta-d-ribofuranoside-5′-monophosphate

## References

[1] Rattigan S, Clark MG, Barrett EJ (1997). Hemodynamic actions of insulin in rat skeletal muscle: evidence for capillary recruitment. Diabetes.

[2] Vincent MA, Dawson D, Clark AD, Lindner JR, Rattigan S, Clark MG (2002). Skeletal muscle microvascular recruitment by physiological hyperinsulinemia precedes increases in total blood flow. Diabetes.

[3] Vincent MA, Clerk LH, Lindner JR, Klibanov AL, Clark MG, Rattigan S (2004). Microvascular recruitment is an early insulin effect that regulates skeletal muscle glucose uptake in vivo. Diabetes.

[4] Vincent MA, Barrett EJ, Lindner JR, Clark MG, Rattigan S (2003). Inhibiting NOS blocks microvascular recruitment and blunts muscle glucose uptake in response to insulin. Am J Physiol Endocrinol Metab.

[5] Rodnick KJ, Henriksen EJ, James DE, Holloszy JO (1992). Exercise training, glucose transporters, and glucose transport in rat skeletal muscles. Am J Physiol.

[6] Stephens JM, Pilch PF (1995). The metabolic regulation and vesicular transport of GLUT4, the major insulin-responsive glucose transporter. Endocr Rev.

[7] Clerk LH, Vincent MA, Jahn LA, Liu Z, Lindner JR, Barrett EJ (2006). Obesity blunts insulin-mediated microvascular recruitment in human forearm muscle. Diabetes.

[8] Keske MA, Clerk LH, Price WJ, Jahn LA, Barrett EJ (2009). Obesity blunts microvascular recruitment in human forearm muscle after a mixed meal. Diabetes Care.

[9] Sjoberg KA, Rattigan S, Hiscock NJ, Richter EA, Kiens B (2011). A new method to study changes in microvascular blood volume in muscle and adipose tissue: real time imaging in humans and rat. Am J Physiol Heart Circ Physiol.

[10] Vincent MA, Clerk LH, Lindner JR, Price WJ, Jahn LA, Leong-Poi H (2006). Mixed meal and light exercise each recruit muscle capillaries in healthy humans. Am J Physiol Endocrinol Metab.

[11] Kubota T, Kubota N, Kumagai H, Yamaguchi S, Kozono H, Takahashi T (2011). Impaired insulin signaling in endothelial cells reduces insulin-induced glucose uptake by skeletal muscle. Cell Metab.

[12] Premilovac D, Bradley EA, Ng HL, Richards SM, Rattigan S, Keske MA (2013). Muscle insulin resistance resulting from impaired microvascular insulin sensitivity in Sprague Dawley rats. Cardiovasc Res.

[13] St Pierre P, Genders AJ, Keske MA, Richards SM, Rattigan S (2010). Loss of insulin-mediated microvascular perfusion in skeletal muscle is associated with the development of insulin resistance. Diabetes Obes Metab.

[14] Wallis MG, Wheatley CM, Rattigan S, Barrett EJ, Clark AD, Clark MG (2002). Insulin-mediated hemodynamic changes are impaired in muscle of zucker obese rats. Diabetes.

[15] Clark MG (2008). Impaired microvascular perfusion: a consequence of vascular dysfunction and a potential cause of insulin resistance in muscle. Am J Physiol Endocrinol Metab.

[16] Scherrer U, Randin D, Vollenweider P, Vollenweider L, Nicod P (1994). Nitric oxide release accounts for insulin’s vascular effects in humans. J Clin Invest.

[17] Steinberg HO, Brechtel G, Johnson A, Fineberg N, Baron AD (1994). Insulin-mediated skeletal muscle vasodilation is nitric oxide dependent. A novel action of insulin to increase nitric oxide release. J Clin Invest..

[18] Bradley EA, Richards SM, Keske MA, Rattigan S (2013). Local NOS inhibition impairs vascular and metabolic actions of insulin in rat hindleg muscle in vivo. Am J Physiol Endocrinol Metab.

[19] Natali A, Quinones GA, Pecori N, Sanna G, Toschi E, Ferrannini E (1998). Vasodilation with sodium nitroprusside does not improve insulin action in essential hypertension. Hypertension.

[20] Mahajan H, Richards SM, Rattigan S, Clark MG (2004). Local methacholine but not bradykinin potentiates insulin-mediated glucose uptake in muscle in vivo by augmenting capillary recruitment. Diabetologia.

[21] Clark MG, Wallis MG, Barrett EJ, Vincent MA, Richards SM, Clerk LH (2003). Blood flow and muscle metabolism: a focus on insulin action. Am J Physiol Endocrinol Metab.

[22] Montagnani M, Ravichandran LV, Chen H, Esposito DL, Quon MJ (2002). Insulin receptor substrate-1 and phosphoinositide-dependent kinase-1 are required for insulin-stimulated production of nitric oxide in endothelial cells. Mol Endocrinol.

[23] Vincent MA, Montagnani M, Quon MJ (2003). Molecular and physiologic actions of insulin related to production of nitric oxide in vascular endothelium. CurrDiabRep.

[24] Bradley EA, Clark MG, Rattigan S (2007). Acute effects of wortmannin on insulin’s hemodynamic and metabolic actions in vivo. Am J Physiol Endocrinol Metab.

[25] Bradley EA, Eringa EC, Stehouwer CD, Korstjens I, Nieuw Amerongen GP, Musters R (2010). Activation of AMP-activated protein kinase by 5-aminoimidazole-4-carboxamide-1-beta-d-ribofuranoside in the muscle microcirculation increases nitric oxide synthesis and microvascular perfusion. Arterioscler Thromb Vasc Biol.

[26] Bergeron R, Russell RR, Young LH, Ren JM, Marcucci M, Lee A (1999). Effect of AMPK activation on muscle glucose metabolism in conscious rats. Am J Physiol.

[27] Fisher JS, Gao J, Han DH, Holloszy JO, Nolte LA (2002). Activation of AMP kinase enhances sensitivity of muscle glucose transport to insulin. Am J Physiol Endocrinol Metab.

[28] Bergeron R, Previs SF, Cline GW, Perret P, Russell RR, Young LH (2001). Effect of 5-aminoimidazole-4-carboxamide-1-beta-d-ribofuranoside infusion on in vivo glucose and lipid metabolism in lean and obese Zucker rats. Diabetes.

[29] Wei K, Jayaweera AR, Firoozan S, Linka A, Skyba DM, Kaul S (1998). Quantification of myocardial blood flow with ultrasound-induced destruction of microbubbles administered as a constant venous infusion. Circulation.

[30] Inyard AC, Clerk LH, Vincent MA, Barrett EJ (2007). Contraction stimulates nitric oxide independent microvascular recruitment and increases muscle insulin uptake. Diabetes.

[31] Wynants J, Petrov B, Nijhof J, Van Belle H (1987). Optimization of a high-performance liquid chromatographic method for the determination of nucleosides and their catabolies. Application to cat and rabbit heart perfusates. J Chromatogr.

[32] Laughlin MH, Armstrong RB (1983). Rat muscle blood flows as a function of time during prolonged slow treadmill exercise. Am J Physiol.

[33] Morrow VA, Foufelle F, Connell JM, Petrie JR, Gould GW, Salt IP (2003). Direct activation of AMP-activated protein kinase stimulates nitric oxide synthesis in human aortic endothelial cells. J Biol Chem.

[34] Iglesias MA, Ye JM, Frangioudakis G, Saha AK, Tomas E, Ruderman NB (2002). AICAR administration causes an apparent enhancement of muscle and liver insulin action in insulin-resistant high-fat-fed rats. Diabetes.

[35] Iglesias MA, Furler SM, Cooney GJ, Kraegen EW, Ye JM (2004). AMP-activated protein kinase activation by AICAR increases both muscle fatty acid and glucose uptake in white muscle of insulin-resistant rats in vivo. Diabetes.

[36] Ai H, Ihlemann J, Hellsten Y, Lauritzen HP, Hardie DG, Galbo H (2002). Effect of fiber type and nutritional state on AICAR- and contraction-stimulated glucose transport in rat muscle. Am J Physiol Endocrinol Metab.

[37] Putman CT, Martins KJ, Gallo ME, Lopaschuk GD, Pearcey JA, MacLean IM (2007). Alpha-catalytic subunits of 5′AMP-activated protein kinase display fiber-specific expression and are upregulated by chronic low-frequency stimulation in rat muscle. Am J Physiol Regul Integr Comp Physiol.

[38] Pencek RR, Shearer J, Camacho RC, James FD, Lacy DB, Fueger PT (2005). 5-Aminoimidazole-4-carboxamide-1-beta-d-ribofuranoside causes acute hepatic insulin resistance in vivo. Diabetes.

[39] Camacho RC, Pencek RR, Lacy DB, James FD, Donahue EP, Wasserman DH (2005). Portal venous 5-aminoimidazole-4-carboxamide-1-beta-d-ribofuranoside infusion overcomes hyperinsulinemic suppression of endogenous glucose output. Diabetes.

[40] Camacho RC, Lacy DB, James FD, Donahue EP, Wasserman DH (2005). 5-Aminoimidazole-4-carboxamide-1-{beta}-d-ribofuranoside renders glucose output by the liver of the dog insensitive to a pharmacological increment in insulin. Am J Physiol Endocrinol Metab.

[41] Kurth-Kraczek EJ, Hirshman MF, Goodyear LJ, Winder WW (1999). 5′ AMP-activated protein kinase activation causes GLUT4 translocation in skeletal muscle. Diabetes.

[42] Merrill GF, Kurth EJ, Hardie DG, Winder WW (1997). AICA riboside increases AMP-activated protein kinase, fatty acid oxidation, and glucose uptake in rat muscle. Am J Physiol.

[43] Holmes BF, Kurth-Kraczek EJ, Winder WW (1999). Chronic activation of 5′-AMP-activated protein kinase increases GLUT-4, hexokinase, and glycogen in muscle. J Appl Physiol.

[44] Winder WW (2000). AMP-activated protein kinase: possible target for treatment of type 2 diabetes. Diabetes Technol Ther.

[45] Atkinson LL, Kozak R, Kelly SE, Onay Besikci A, Russell JC, Lopaschuk GD (2003). Potential mechanisms and consequences of cardiac triacylglycerol accumulation in insulin-resistant rats. Am J Physiol Endocrinol Metab.

[46] Halse R, Fryer LG, McCormack JG, Carling D, Yeaman SJ (2003). Regulation of glycogen synthase by glucose and glycogen: a possible role for AMP-activated protein kinase. Diabetes.

[47] Hue L, Beauloye C, Marsin AS, Bertrand L, Horman S, Rider MH (2002). Insulin and ischemia stimulate glycolysis by acting on the same targets through different and opposing signaling pathways. J Mol Cell Cardiol.

[48] Longnus SL, Wambolt RB, Parsons HL, Brownsey RW, Allard MF (2003). 5-Aminoimidazole-4-carboxamide 1-beta-d-ribofuranoside (AICAR) stimulates myocardial glycogenolysis by allosteric mechanisms. Am J Physiol Regul Integr Comp Physiol.

[49] Jung CH, Lee MJ, Kang YM, Lee YL, Yoon HK, Kang SW (2014). Vaspin inhibits cytokine-induced nuclear factor-kappa B activation and adhesion molecule expression via AMP-activated protein kinase activation in vascular endothelial cells. Cardiovasc Diabetol.

[50] Anil TM, Harish C, Lakshmi MN, Harsha K, Onkaramurthy M, Sathish Kumar V (2014). CNX-012-570, a direct AMPK activator provides strong glycemic and lipid control along with significant reduction in body weight; studies from both diet-induced obese mice and db/db mice models. Cardiovasc Diabetol.

[51] Clerk LH, Rattigan S, Clark MG (2002). Lipid infusion impairs physiologic insulin-mediated capillary recruitment and muscle glucose uptake in vivo. Diabetes.

[52] Wheatley CM, Rattigan S, Richards SM, Barrett EJ, Clark MG (2004). Skeletal muscle contraction stimulates capillary recruitment and glucose uptake in insulin-resistant obese Zucker rats. Am J Physiol Endocrinol Metab.

[53] Inyard AC, Chong DG, Klibanov AL, Barrett EJ (2009). Muscle contraction, but not insulin, increases microvascular blood volume in the presence of free fatty acid-induced insulin resistance. Diabetes.

[54] Lee-Young RS, Bonner JS, Mayes WH, Iwueke I, Barrick BA, Hasenour CM (2013). AMP-activated protein kinase (AMPK)alpha2 plays a role in determining the cellular fate of glucose in insulin-resistant mouse skeletal muscle. Diabetologia.

